# A clinical prediction model for delirium tremens: development and validation in alcohol-dependent patients using multivariable logistic regression

**DOI:** 10.3389/fpsyt.2026.1712870

**Published:** 2026-02-27

**Authors:** Jing Zhong, Xiaoyu Huang, Xudong Yao

**Affiliations:** The Forth People’s Hospital of Chengdu, Chengdu, China

**Keywords:** alcohol dependence, clinical prediction model, delirium tremens, DT, logistic regression

## Abstract

**Background:**

Delirium tremens (DT) is a severe complication of alcohol withdrawal. This study aimed to develop and validate a prediction model for DT risk in hospitalized patients with alcohol dependence, using routine laboratory indicators.

**Methods:**

We retrospectively analyzed 347 patients with alcohol dependence admitted to the Addiction Medicine Department of a tertiary psychiatric hospital from 2020 to 2024. The primary outcome was DT occurrence. A prediction model was constructed using logistic regression, with data split into training (70%) and validation (30%) sets by random sampling. Model performance was evaluated via the area under the receiver operating characteristic curve (AUC), calibration plots, and decision curve analysis (DCA).

**Results:**

Of 347 patients, 118 (34%) developed DT. LASSO regression identified 11 predictors: history of DT, ammonia, creatinine, uric acid, total bilirubin (Tbiliary), albumin (ALB), gamma-glutamyl transferase (GGT), chloride (Cl), free triiodothyronine (Free_T3), free thyroxine (Free_T4), neutrophil percentage (NEU%), and red blood cell (RBC) count. Logistic regression confirmed that history of DT, ammonia, creatinine, ALB, Free_T3, NEU%, and RBC were independent risk factors (P< 0.05). The model demonstrated robust performance: AUC = 0.9881 [95% CI: 0.9794–0.9967] in the training set and 0.9599 [95% CI: 0.9142–1.0000] in the validation set, with high net benefit in DCA.

**Conclusions:**

This model, incorporating readily available biomarkers and clinical history, effectively predicts DT risk. Limitations include its retrospective design (potential selection bias) and exclusion of clinical scales (e.g., CIWA-Ar). Prospective multicenter studies are needed to validate its generalizability.

## 1 Introduction

Alcohol withdrawal syndrome (AWS) is a clinically significant constellation of symptoms occurring in alcohol-dependent individuals following abrupt cessation or reduction of alcohol intake, characterized by ≥2 features including autonomic hyperactivity (e.g., sweating, heart rate >100 bpm), exacerbated tremors, insomnia, nausea/vomiting, hallucinations or illusions, psychomotor agitation, anxiety, and generalized tonic-clonic seizures, which collectively lead to impaired social or occupational functioning (1, 2). Among AWS subtypes, delirium tremens (DT) represents the most severe manifestation, affecting 5–20% of withdrawal cases and typically manifesting 48–72 hours after the last drink, with a duration of 1–8 days (3, 4). Despite its high mortality risk (up to 15–20% in untreated cases), timely intervention with benzodiazepines can reduce mortality to<1% (5). However, DT progresses rapidly and may induce fatal complications (e.g., arrhythmias, hyperthermia, or respiratory failure), making early identification of high-risk individuals critical for preventive interventions.

Accumulating evidence underscores the role of systemic inflammation and immune activation in the pathophysiology of DT. Recent studies have explored novel hematological and biochemical markers for risk stratification. Notably, the neutrophil-to-lymphocyte ratio (NLR), an easily calculable marker of systemic inflammation, has shown promise in predicting the development and severity of DT. Elevated NLR reflects a state of neutrophilia and relative lymphopenia, indicative of heightened stress and inflammatory response during alcohol withdrawal (6). The parameters selected for investigation in this study—encompassing liver function, renal function, electrolytes, thyroid hormones, and comprehensive hematologic indices—were chosen based on their established or postulated links to the multifaceted pathophysiology of AWS and DT. These include direct organ damage from chronic alcohol use (liver, kidney), metabolic disturbances, and neuroendocrine-immune interactions that modulate neuronal excitability (7).

Existing evidence on patient-level DT risk factors remains limited due to small sample sizes in prior studies, failure to adjust for confounding variables, and inconsistent findings across cohorts. While clinical guidelines emphasize benzodiazepine prophylaxis, no widely validated, bedside-accessible prediction tool exists to stratify DT risk based on routinely available biomarkers and clinical data. To address this gap, we aimed to develop and validate a predictive model integrating both established (e.g., history of DT) and acute measurable indicators (e.g., laboratory parameters). This tool is designed to enhance early risk stratification, guide personalized benzodiazepine protocols, and reduce preventable morbidity/mortality, thereby translating empirical evidence into a clinically actionable solution for AWS management.

## 2 Objects and methods

### 2.1 Research objects

In this retrospective study, we included patients with alcohol dependence who were hospitalized in the Department of Addiction Medicine of Chengdu Fourth People’s Hospital from January 2020 to December 2024. Inclusion criteria: (1) Alcohol-dependent patients who meet the ICD-10 diagnostic criteria. Exclusion Criteria: Combined with other psychoactive substance abuse.

### 2.2 Data integration

The study dataset comprised comprehensive clinical and laboratory information from patients with alcohol dependence. The outcome variable was binary, classifying patients as either presenting with delirium tremens (DT) or not.

Patient baseline characteristics included:

- Demographic variables: age, drinking duration (years), and daily alcohol consumption
- Clinical history: body mass index (BMI), history of delirium tremens
- Chronic conditions: presence/absence of hypertension, diabetes mellitus, and cardiovascular disease

Laboratory parameters encompassed:

- Hepatic function markers: blood ammonia, total bilirubin, direct bilirubin (Tbiliary), albumin (ALB), aspartate aminotransferase (AST), alanine aminotransferase (ALT), gamma-glutamyl transferase (GGT)
- Renal function markers: blood urea nitrogen, creatinine, urea
- Electrolytes: potassium (K+), sodium (Na+), chloride (Cl-)
- Thyroid function: free triiodothyronine (Free_T3), free thyroxine (Free_T4), thyroid-stimulating hormone (TSH)
- Hematologic parameters: white blood cell count (WBC), neutrophil count, lymphocyte count, neutrophil percentage (NEU%), lymphocyte percentage, red blood cell (RBC) count, hemoglobin (HGB), platelet count
- Inflammatory marker: C-reactive protein (CRP)

All laboratory measurements were obtained from standard blood tests performed at hospital admission. Missing data were handled according to predefined criteria (see Supplementary Methods).

### 2.3 Statistical methods

All analyses were done in R 4.4.3. Continuous variables are expressed as means± standard deviations or medians and ranges based on the degree of contribution of the data. Categorical variables are expressed as frequencies and percentages (%). We used unpaired t-test, Wilcoxon rank-sum test, Pearson chi-square test, or Fisher’s exact test to compare between groups. ROC curves were plotted and AUC was calculated to assess univariate ability to predict delirium tremens. LASSO regression (10-fold cross-validation to determine the optimal λ) was applied to shrink the partial coefficients to 0, and variable selection and model dimensionality reduction were realized (8). The Logistic regression model was constructed with the variables retained by LASSO and a nomogram was plotted (9). The receiver operating characteristic curve (ROC curve) was used to evaluate the prediction performance of the model, and the larger the area under the curve (AUC), the stronger the discriminant ability of the model. In general, an AUC of 0.5 suggests no discrimination (i.e., ability to diagnose patients with and without the disease or condition based on the test), 0.7 to 0.8 is considered acceptable, 0.8 to 0.9 is considered excellent, and more than 0.9 is considered outstanding (10). The nomogram was verified by the Bootstrap method (the number of samplings was set to 1–000 times), and the calibration curve was drawn to evaluate the calibration degree of the model, and the closer the calibration curve was to the diagonal, the better the calibration degree of the model (11). Decision curve analysis (DCA) was used to evaluate the clinical net benefits of three strategies (predictive model, treat all patients, and no treatment) under different risk thresholds (12).

## 3 Results

### 3.1 Patient characteristics

A total of 347 patients with alcohol dependence were included, of which 118 had delirium tremens, with an incidence rate of 34%. There were significant differences in age, BMI, blood ammonia, alcohol consumption, urea nitrogen, creatinine, urea, total bilirubin, albumin, ALT, AST, GGT, sodium, potassium, Free_T3, Free_T4, history of delirium tremens, WBC, NEU, LYM, NEU%, LYM%, RBC, HGB, and PLT between the two groups (P<0.05). There were no significant differences in years of drinking, chloride concentration, TSH, comparison of underlying diseases, and C-reactive protein between the two groups (P>0.05). The results are shown in **Table 1**.

**Table 1:** Patient characteristics.

Variable	Non-delirium tremens (n=229)	delirium tremens (n=118)	P
BMI (kg/m²)	17.6 (16, 19.1)	18.5 (16.42, 19.28)	0.007
Age (years)	50 (43, 56)	54 (45, 57.75)	0.031
Years of drinking (years)	21 (15, 30)	20 (13, 30)	0.448
chronic diseases NO YES	189 (83) 40 (17)	100 (85) 18 (15)	0.71
Alcohol Consumption (g)	450 (350, 500)	450 (400, 500)	0.022
Ammonia (μmol/L)	36.6 (23.7, 44.5)	47.6 (39.58, 56.7)	<0.001
BUN (mmol/L)	3.3 (2.4, 4.2)	3.8 (2.85, 5.9)	0.003
Creatinine (μmol/L)	63 (55, 73)	75 (55.25, 101.25)	<0.001
Uric acid (μmol/L)	455 (352, 532)	371.5 (311, 485)	0.002
Total bilirubin (U/L)	14 (9.5, 19.2)	17.95 (11.6, 26.97)	<0.001
Albumin (g/L)	41.5 (39.5, 44.9)	32.3 (26.92, 41.5)	<0.001
ALT (U/L)	33 (20, 52)	42.5 (29, 54.75)	0.004
AST (U/L)	50 (36, 58)	78 (27, 103)	0.023
GGT (U/L)	139 (94, 214)	146.5 (97.25, 356)	0.049
K- (mmol/L)	3.73 (3.46, 3.86)	3.46 (3.1, 3.86)	0.004
Na-(mmol/L)	141.8 (140.3, 145.1)	141.1 (137.8, 143.7)	0.013
CL-(mmol/L)	103.4 (100.8, 106.3)	102.9 (99.4, 105.7)	0.065
Free T3 (pmol/L)	4.73 (4.13, 5.2)	5.64 (4.68, 6.38)	<0.001
Free T4 (pmol/L)	10.29 (9.16, 12.07)	13.72 (10.09, 20.1)	<0.001
TSH (mIU/L)	1.56 (1.07, 2.63)	1.78 (1.17, 2.65)	0.314
Leukocytes (×10^9^/L)	7.13 (5.02, 8.61)	7.74 (6.21, 8.34)	0.023
Neutrophils (×10^9^/L)	4.77 (2.81, 6.34)	6.49 (4.71, 10.18)	<0.001
Lymphocytes (×10^9^/L)	1.69 (1.08, 2.13)	1.21 (0.86, 1.95)	<0.001
Neutrophil ratio(%)	62.8 (56.4, 71.7)	78 (70.2, 84.4)	<0.001
Lymphocyte ratio (%)	20.1 (10.9, 29)	13.8 (6.18, 20.4)	<0.001
Redbloodcells(×10¹²/L)	4.6 (4.13, 5.34)	4.13 (3.43, 4.44)	<0.001
hemoglobin(g/L)	142 (128, 154)	134 (120, 142)	0.003
platelets(×10^9^/L)	183 (127, 243)	144 (98.25, 212)	0.002
C-reactive protein (mg/L)	4.61 (1.61, 9.9)	5.41 (0.9, 27.03)	0.078
Previous DT NO YES	205 (90) 24 (10)	65 (55) 53 (45)	<0.001

1. Categorical variables are expressed as frequencies (percentages), continuous variables are expressed as medians (first quartile, third quartile) 2. All units are marked in parentheses after variable names 3. P values< 0.05 are considered statistically significant.

### 3.2 Univariate ROC analysis

**Figure 1:**
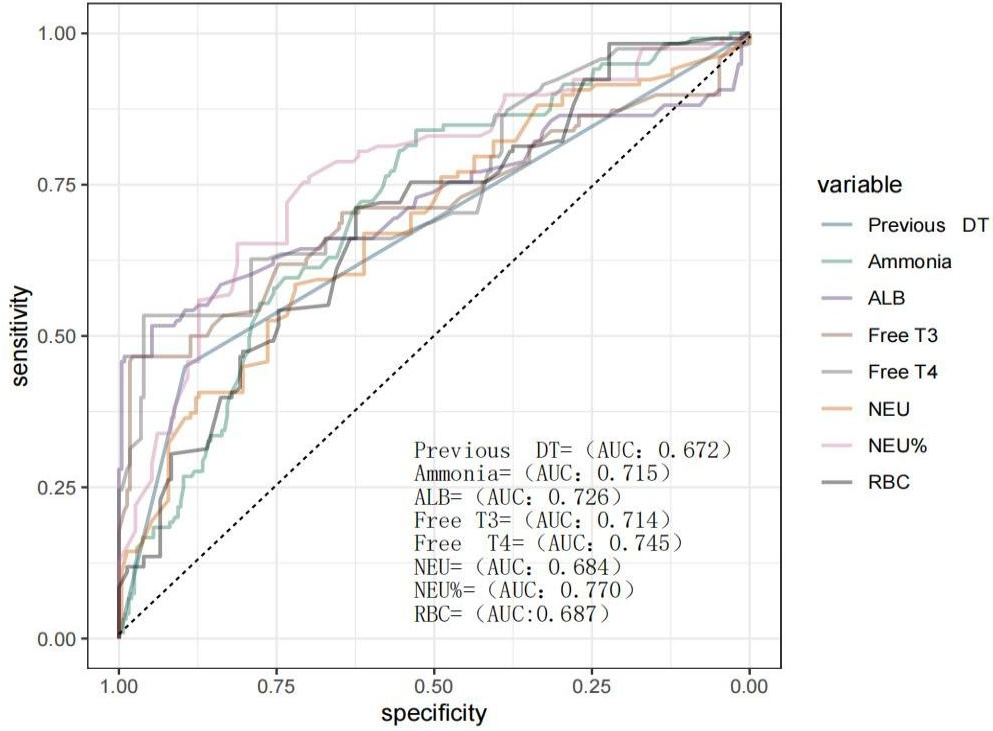
ROC curves for predicting delirium tremens.

The predictive performance of individual clinical indicators for delirium tremens (DT) was evaluated by constructing univariate ROC curves. The results demonstrated moderate predictive value (AUC > 0.6) for the following variables: neutrophil percentage (AUC = 0.77, 95% CI: [0.72-0.82]), absolute neutrophil count (AUC = 0.68, 95% CI: [0.62-0.74]), free thyroxine (Free_T4; AUC = 0.74, 95% CI: [0.68-0.80), free triiodothyronine (Free_T3; AUC = 0.71, 95% CI: [0.65-0.78]), serum albumin (AUC = 0.73, 95% CI: [0.66-0.79]), blood ammonia (AUC = 0.71, 95% CI: [0.66-0.77]), hemoglobin (AUC = 0.69, 95% CI: [0.63-0.75]), and history of DT (AUC = 0.67, 95% CI: [0.62-0.72]). These findings suggest that these parameters exhibit moderate discriminatory ability for DT risk. When the 95% confidence interval of the AUC does not include 0.5, it generally implies that the p-value is less than 0.05, indicating statistical significance against the null hypothesis of no discriminative power (AUC = 0.5) (13). The ROC curves are presented in **Figure 1**.

### 3.3 Variable selection using LASSO regression

**Figure 2:**
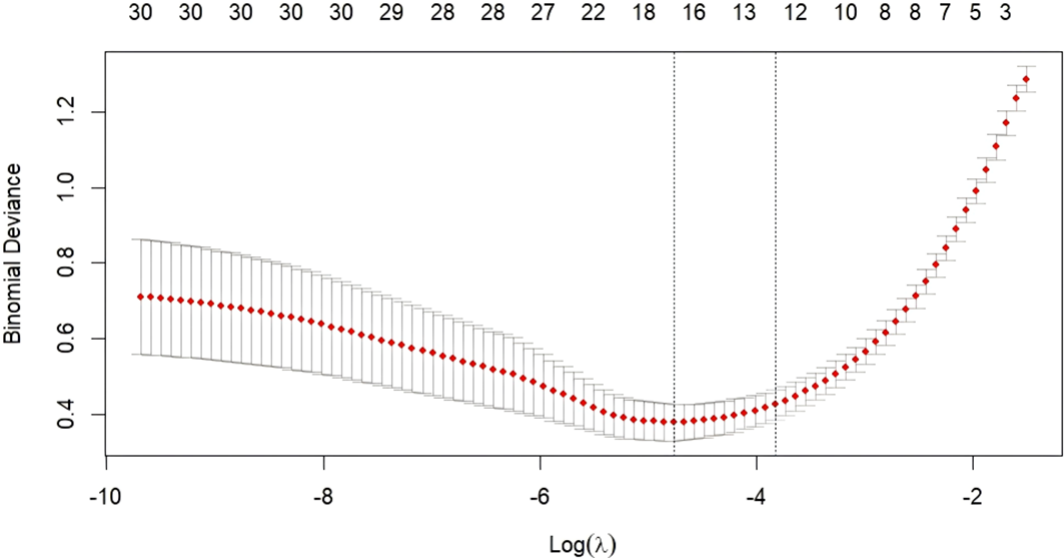
LASSO coefficient profiles and cross-validation for variable selection.

LASSO (Least Absolute Shrinkage and Selection Operator) regression was employed to identify optimal predictors from the training dataset. Two critical regularization parameters were evaluated: Lambda.min (λ=0.008) and Lambda.1se (λ=0.018), represented by two vertical dashed lines in **Figure 2**. When Lambda.min was applied, 18 variables were retained; in contrast, Lambda.1se - a more parsimonious criterion balancing bias-variance tradeoff - selected 12 predictors. The final prediction model was constructed using the 12 variables identified at Lambda.1se: history of delirium tremens (DT), blood ammonia, creatinine, uric acid, total bilirubin (Tbiliary), albumin (ALB), gamma-glutamyl transferase (GGT), chloride (Cl^−^), free triiodothyronine (Free_T3), free thyroxine (Free_T4), neutrophil percentage (NEU%), and red blood cell (RBC) count.

### 3.4 Multivariable logistic regression analysis

A multivariable logistic regression model was constructed to identify independent predictors of delirium tremens (DT). As shown in **Table 2**, seven variables demonstrated statistically significant associations with DT incidence (P< 0.05): history of DT, blood ammonia, creatinine, albumin, free triiodothyronine (Free_T3), neutrophil percentage (NEU%), and red blood cell (RBC) count.

**Table 2:** Multivariable logistic regression analysis of predictors for delirium tremens.

Variable	Coef	S.E.	Wald Z​	​P	OR (95% CI)​​
Previous DT	2.5517	0.9328	2.74	0.006	12.82 (2.08–78.31)
ammonia	0.0524	0.0216	2.42	0.016	1.05 (1.01–1.10)
Tbiliary	0.0334	0.0174	1.92	0.055	1.03(0.99-1.06)
Uric acid	0.0001	0.0027	0.03	0.978	1.00 (0.99–1.00)
Creatinine	0.0924	0.0226	4.10	<0.001	1.10 (1.05–1.15)
GGT	-0.0002	0.0016	-0.10	0.919	1.00 (0.99–1.00)
ALB	-0.5301	0.1243	-4.27	<0.001	0.59 (0.46–0.75)
Cl^−^	-0.0555	0.0664	-0.84	0.404	0.95 (0.83–1.08)
Free_T3	1.5461	0.4294	3.60	0.0003	4.70 (2.02–10.94)
Free_T4	-0.0717	0.1385	-0.52	0.605	0.93 (0.71–1.22)
NEU%	0.1961	0.0576	3.40	<0.001​	1.22 (1.09–1.37)
RBC	-2.1887	0.6485	-3.37	<0.001​	0.11 (0.03–0.38)

DT, delirium tremens; S.E., standard error; OR, odds ratio; CI, confidence interval.

Statistical significance: P< 0.05; ​P< 0.01; ​​P< 0.001.

**Figure 3:**
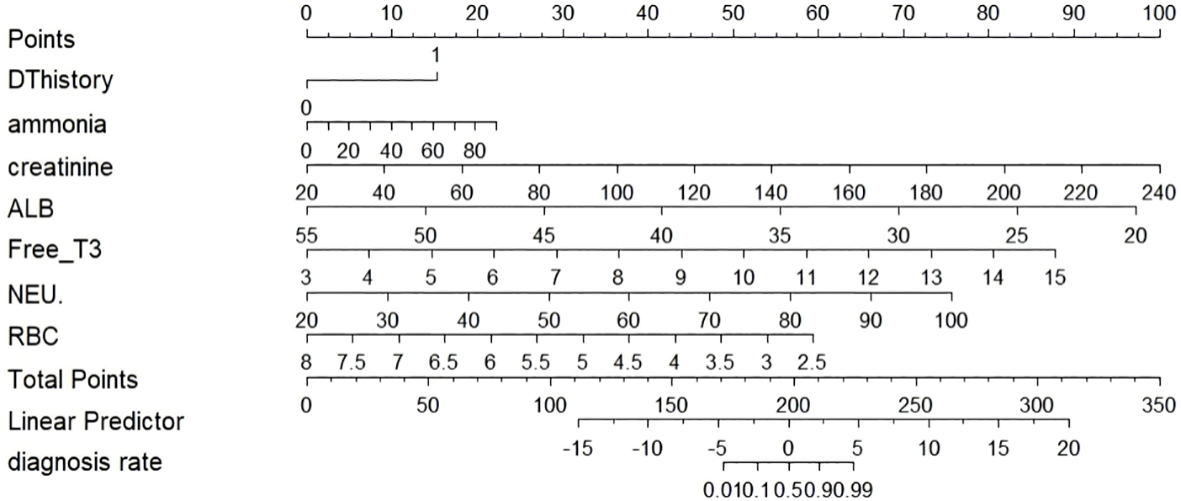
Nomogram for predicting delirium tremens (DT) risk.

Based on these findings, a predictive model was established using the rms package in R software (version 4.4.3). The logistic regression equation was fitted with the following form:


$$\begin{array}{c} \backslash \text{logit}\left(\text{P}\right)=\beta 0+\beta 1\left(\text{History of DT}\right)+\beta 2\left(\text{Ammonia}\right)+\beta 3\left(\text{Creatinine}\right)\\ +\beta 4\left(\text{Albumin}\right)+\beta 5\left(Free\_T3\right)+\beta 6\left(\text{NEU\%}\right)+\beta 7\left(\text{RBC}\right) \end{array}$$

where β represents the regression coefficients for each predictor. The model’s clinical utility was visualized by constructing a nomogram (**Figure 3**), which integrates the contributions of all significant predictors to provide individualized DT risk scores.

### 3.5 Model validation

The model’s performance was comprehensively evaluated through discrimination and calibration analyses.

#### 3.5.1 Discrimination ability

**Figure 4:**
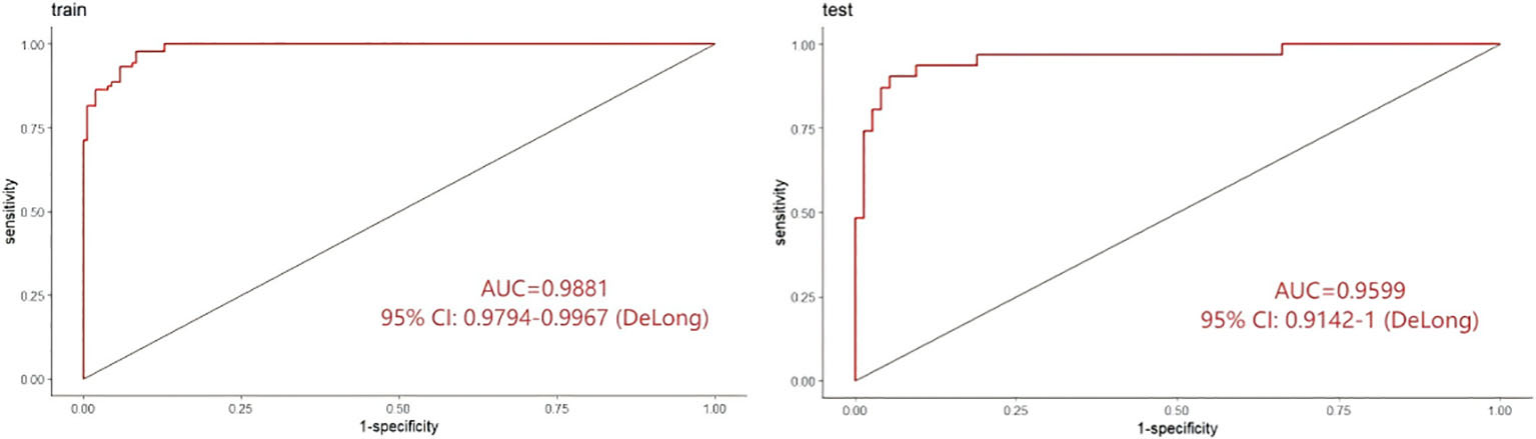
ROC curves of the prediction model for delirium tremens (DT).

The receiver operating characteristic (ROC) curve analysis demonstrated excellent discriminative power for both the training and validation datasets. The area under the ROC curve (AUC) was ​0.9881​ [95% confidence interval (CI): 0.9794–0.9967] in the training set and ​0.9599​ [95% CI: 0.9142–1.0000] in the validation set, indicating robust predictive capability across datasets (**Figure 4**).

#### 3.5.2 Calibration accuracy

**Figure 5:**
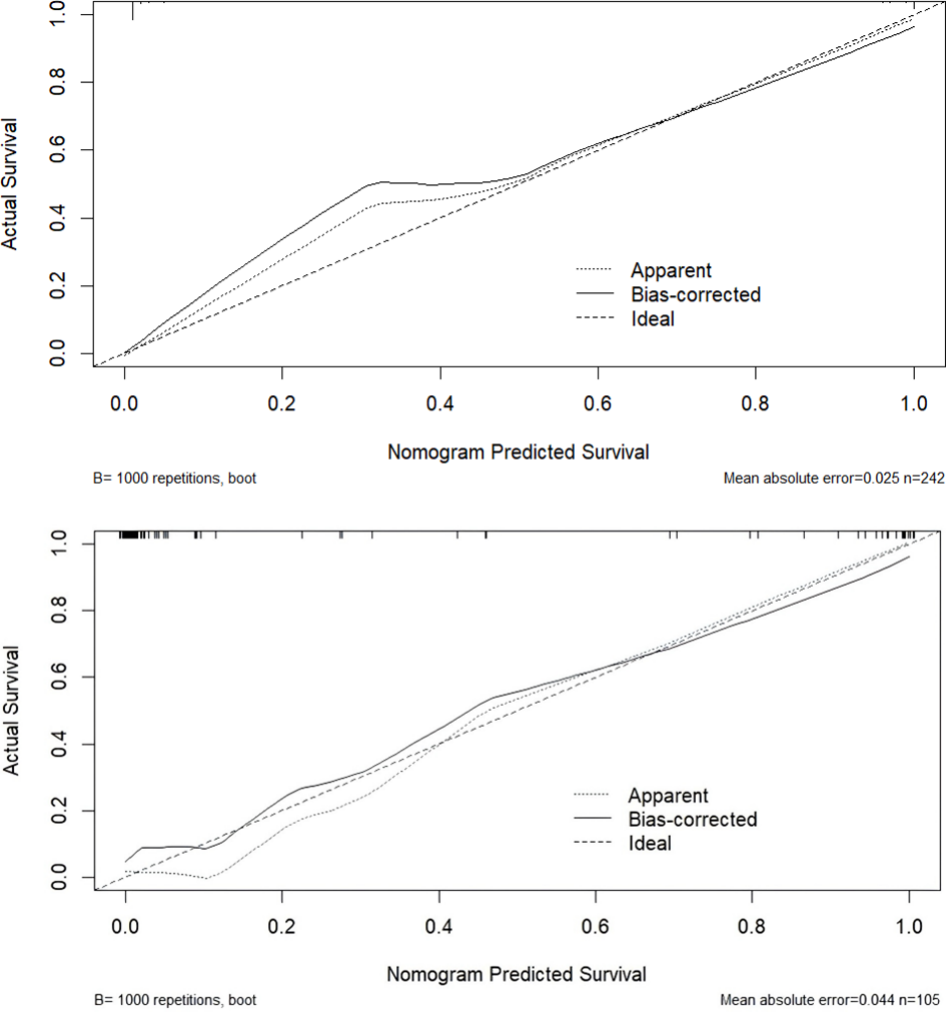
Calibration curve of the delirium tremens (DT) prediction model.

Calibration was assessed using the Bootstrap method (1,000 resamples) to generate calibration curves. The model’s predicted probabilities showed strong agreement with observed outcomes, as evidenced by the close alignment of the calibration curve with the ideal 45° diagonal line (**Figure 5**). This indicates high calibration accuracy and reliable probability estimates.

### 3.6 Clinical utility evaluation via decision curve analysis

**Figure 6:**
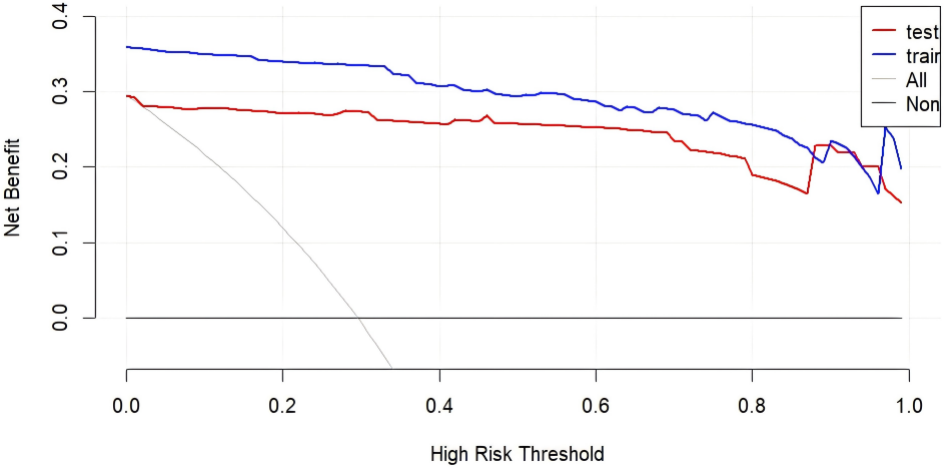
Decision curve analysis (DCA) of the delirium tremens (DT) prediction model.

Decision curve analysis (DCA) was performed to assess the clinical applicability of the prediction model. The results demonstrated that the model provided substantial net benefit across a clinically relevant range of threshold probabilities. Specifically, in the training set, the model exhibited high net benefit when the threshold probability for DT occurrence ranged from ​0.1 to 0.2. Similarly, in the validation set, the highest net benefit was observed when the threshold probability ranged from ​0.05 to 0.2​ (**Figure 6**). These findings indicate that the model is clinically useful for identifying high-risk patients within these probability thresholds, supporting its potential integration into routine clinical workflows.

## 4 Discussion

Alcohol, a central nervous system (CNS) depressant, is a lipophilic and neurotropic substance that exerts direct neurotoxic effects on the human brain. During alcohol intoxication, the permeability of the blood-brain barrier increases, leading to extensive and severe damage to the CNS, which may result in cerebral atrophy. Upon cessation of alcohol consumption, individuals may experience withdrawal syndrome, with severe cases presenting as delirium tremens. Under conditions of excessive alcohol intake, ethanol markedly enhances the activation of GABA receptors, thereby potentiating neuronal inhibition (14, 15). As excessive drinking continues, tolerance develops, necessitating greater amounts of alcohol to sustain GABA-mediated inhibitory effects. Concurrently, glutamate production increases to compensate for the heightened inhibition. When alcohol is discontinued during withdrawal, the inhibitory stimulation via GABA ceases. However, excess glutamate affects NMDA receptors, overloading neurons with excitatory neurotransmission (16). This elevates the likelihood of neuronal cell death due to excitotoxicity (17). Clinical signs and symptoms include palpitations, anxiety, tremors, headaches, restlessness, and seizures (18, 19).

In this retrospective cohort study aimed at identifying clinical predictors of delirium tremens (DT) in alcohol dependence and evaluating their utility, we observed that DT developed in 34% of the cases diagnosed with alcohol dependence. This incidence is substantially higher than that reported in previous studies. Furthermore, no mortality occurred among the DT patients in our cohort. The increased survival rate may be attributable to the timely administration of benzodiazepines and the active correction of acid-base imbalances and fluid-electrolyte disturbances (20). However, a larger sample size is required to more accurately determine the prevalence of DT and its associated mortality rate.

In this cohort study, comparative analysis of clinical characteristics between the delirium tremens (DT) group and the non-DT group revealed that the DT group had significantly more abnormalities in liver function, renal function, electrolyte levels, and complete blood count parameters. These findings suggest that patients who develop DT are in poorer physical condition compared to those who do not. The pathophysiology of DT involves a series of neurochemical imbalances triggered by abrupt alcohol withdrawal. Long-term alcohol dependence leads to adaptive changes in the central nervous system (CNS), resulting in dysregulation of the neurotransmitters gamma-aminobutyric acid (GABA) and glutamate (21). Alcohol, as a central nervous system depressant, acts by downregulating GABA receptors and upregulating N-methyl-D-aspartate (NMDA) receptor expression, ultimately leading to increased glutamate activity in the CNS. The imbalance between GABAergic inhibition and glutamatergic excitation contributes to the hyperexcitability observed in DT. Excessive glutamate release and subsequent overactivation of NMDA receptors can induce excitotoxicity, causing neuronal damage and exacerbating DT symptoms such as confusion, hallucinations, and tremors (22). Although the exact pathophysiological mechanisms underlying this association remain speculative and require further investigation, patients with a prior history of DT are at significantly increased risk of recurrence. This observation is consistent with previous studies suggesting a “kindling” effect in DT occurrence. The mechanism may involve neuroadaptive changes from initial episodes, such as impaired GABAergic inhibition and glutamatergic hyperactivation, which can be reinforced during subsequent withdrawal periods, thereby increasing relapse risk (23). For instance, Mohan found that patients with a history of DT had a higher likelihood of recurrence, often necessitating ICU management (24). Therefore, in clinical practice, individuals with previous DT should be considered high-risk and require intensified monitoring and intervention during withdrawal.

Denk et al. indicated that liver disease—manifested as increased liver stiffness, elevated bilirubin, and hyperammonemia—is a risk factor for ICU delirium (25). Tobias found that increased liver stiffness is a risk factor for delirium tremens (26), Elevated blood ammonia, reflecting impaired ammonia metabolism due to liver dysfunction, is an important contributor to DT onset. Hildenbrand et al. observed that patients with acute renal failure had a significantly increased risk of delirium (27). Elevated creatinine levels, indicating renal dysfunction and subsequent toxin accumulation, may exacerbate central nervous system toxicity and precipitate tremors (28). Low albumin levels may worsen neurological excitability through poor nutritional status and increased free drug concentrations (20–30). Elevated Free-T3 levels might aggravate withdrawal symptoms by enhancing sympathetic activity and neurotransmitter release. An elevated neutrophil ratio suggests that systemic inflammation contributes to DT via neuroinflammatory pathways. These findings highlight the critical roles of metabolic disturbances, endocrine dysfunction, and inflammatory states in the pathogenesis of DT, offering multi-dimensional perspectives for clinical risk assessment. Future prospective studies are needed to validate the dynamic changes in these biomarkers and to develop individualized prediction models based on multifactor approaches. Such tools could guide early interventions—such as nutritional support, thyroid function monitoring, and liver protection—to reduce the incidence of DT.

In a meta-analysis, hypokalemia and thrombocytopenia were identified as risk factors for the development of severe alcohol withdrawal syndrome (31). The present study primarily included patients with severe alcohol withdrawal requiring hospitalization. This population generally exhibits long-term malnutrition, impaired liver function, and electrolyte disturbances. As a result, the incidence of hypokalemia and thrombocytopenia was notably high and relatively concentrated in this cohort. It should be noted that this study relied on baseline laboratory data obtained at admission. Since the onset of DT is a dynamic process, a single measurement may not adequately capture the fluctuating nature of serum potassium levels or platelet counts, which could attenuate the observed association between these parameters and the actual occurrence of DT.

## 5 Limitations

This study has several limitations: (1) The retrospective design may introduce selection bias; (2) Genetic factors, such as GABA receptor gene polymorphisms, were not considered; (3) The sample was drawn from a single center, which may limit the generalizability of the findings.

## 6 Conclusions

This study identified multiple factors associated with the development of delirium tremens (DT), including a prior history of DT, elevated blood ammonia and creatinine levels, decreased albumin, increased free T3, elevated neutrophil ratio, and red blood cell count. These findings underscore the significance of metabolic abnormalities, previous DT episodes, and thyroid function in assessing DT risk. However, the underlying mechanisms of DT remain incompletely understood. Future longitudinal studies are warranted to validate the dynamic changes in these biomarkers and to develop individualized prediction models based on multifactor approaches for early intervention in high-risk populations.

## Data Availability

The raw data supporting the conclusions of this article will be made available by the authors, without undue reservation.
